# The Effect of Sea Buckthorn (*Hippophae rhamnoides* L.) Seed Oil on UV-Induced Changes in Lipid Metabolism of Human Skin Cells

**DOI:** 10.3390/antiox7090110

**Published:** 2018-08-23

**Authors:** Agnieszka Gęgotek, Anna Jastrząb, Iwona Jarocka-Karpowicz, Marta Muszyńska, Elżbieta Skrzydlewska

**Affiliations:** Department of Inorganic and Analytical Chemistry, Medical University of Bialystok, Bialystok 15-089, Poland; agnieszka.gegotek@umb.edu.pl (A.G.); anna.jastrzab@umb.edu.pl (A.J.); iwona.jarocka-karpowicz@umb.edu.pl (I.J.-K.); marta.muszynska@umb.edu.pl (M.M.)

**Keywords:** fibroblasts, keratinocytes, sea buckthorn seeds oil, UV radiation, lipid metabolism

## Abstract

Lipids and proteins of skin cells are the most exposed to harmful ultraviolet (UV) radiation contained in sunlight. There is a growing need for natural compounds that will protect these sensitive molecules from damage, without harmful side effects. The aim of this study was to investigate the effect of sea buckthorn seed oil on the redox balance and lipid metabolism in UV irradiated cells formed different skin layers to examine whether it had a protective effect. Human keratinocytes and fibroblasts were subjected to UVA (ultraviolet type A; 30 J/cm^2^ and 20 J/cm^2^) or UVB (ultraviolet type B; 60 mJ/cm^2^ and 200 mJ/cm^2^, respectively) radiation and treated with sea buckthorn seed oil (500 ng/mL), and the redox activity was estimated by reactive oxygen species (ROS) generation and enzymatic/non-enzymatic antioxidants activity/level (using electron spin resonance (ESR), high-performance liquid chromatography (HPLC), and spectrophotometry). Lipid metabolism was measured by the level of fatty acids, lipid peroxidation products, endocannabinoids and phospholipase A2 activity (GC/MS (gas chromatography/mass spectrometry), LC/MS (liquid chromatography/mass spectrometry), and spectrophotometry). Also, transcription factor Nrf2 (nuclear erythroid 2-related factor) and its activators/inhibitors, peroxisome proliferator-activated receptors (PPAR) and cannabinoid receptor levels were measured (Western blot). Sea buckthorn oil partially prevents UV-induced ROS generation and enhances the level of non-enzymatic antioxidants such as glutathione (GSH), thioredoxin (Trx) and vitamins E and A. Moreover, it stimulates the activity of Nrf2 leading to enhanced antioxidant enzyme activity. As a result, decreases in lipid peroxidation products (4-hydroxynonenal, 8-isoprostaglandin) and increases in the endocannabinoid receptor levels were observed. Moreover, sea buckthorn oil treatment enhanced the level of phospholipid and free fatty acids, while simultaneously decreasing the cannabinoid receptor expression in UV irradiated keratinocytes and fibroblasts. The main differences in sea buckthorn oil on various skin cell types was observed in the case of PPARs—in keratinocytes following UV radiation PPAR expression was decreased by sea buckthorn oil treatment, while in fibroblasts the reverse effect was observed, indicating an anti-inflammatory effect. With these results, sea buckthorn seed oil exhibited prevention of UV-induced disturbances in redox balance as well as lipid metabolism in skin fibroblasts and keratinocytes, which indicates it is a promising natural compound in skin photo-protection.

## 1. Introduction

Skin has been recognized as an organ that performs synthesis and processing of an astounding range of structural proteins, lipids, and signaling molecules. Moreover, skin is an integral component of the immune, nervous and endocrine systems, and therefore is also responsible for the first line of information and defense in the immunity process [1]. However, skin also protects the organism against the influence of the environment and is well-known as crucial for the maintenance of temperature, electrolyte and fluid balance [2]. As a natural biological barrier, skin is constantly exposed to numerous external factors that can impair the functioning of this barrier, including ultraviolet (UV) radiation contained in sunlight. High energy UVB (ultraviolet type B; 280–320 nm) and UVA (ultraviolet type A; 320–400 nm) radiation absorption by the skin may trigger mechanisms that defend skin integrity and also induces skin pathology, such as cancer [3]. These effects occurs as a result of the UV electromagnetic energy transduction into chemical, hormonal, and neural signals, defined by the nature of the chromophores and tissue compartments receiving specific UV wavelength. However, despite of the wavelength differences, both of them are characterized by high penetration into the deep layers of the epidermis and dermis [4]. UV radiation can upregulate local cytokines, corticotropin-releasing hormone, urocortins, proopiomelanocortin peptides expression, however, they can be released into circulation to exert systemic effects, including activation of the central hypothalamic-pituitary-adrenal axis, opioidogenic effects, and immunosuppression [5]. Similar effects of UV radiation on human organism are seen after exposure of the eyes and skin to UV, through which UV exerts very rapid stimulatory effects on the brain and central neuroendocrine system and impairs body homeostasis [5]. Moreover, UV radiation promotes the generation of reactive oxygen species (ROS) as well as impairs the antioxidant capacity of skin cells [6]. As a result, the redox imbalance leading to oxidative stress is increased. UV-induced oxidative stress in skin enhances the collagen degradation and elastin modification causes premature skin aging [7,8]. Moreover, increased oxidative phospholipid metabolism disturbs the function of cellular membranes, and promotes the generaton of reactive electrophiles, such as 4-hydroxyalkenals [9]. These reactive electrophiles interact with membrane phospholipids and proteins, including receptors, causing irreparable skin damage. Additionally, UV-induced oxidative stress affects phospholipid metabolism which influences the endocannabinoid system [10]. The main endocannabinoids, such as anandamide and 2-arachidonoylglycerol (2-AG), participate in cell signalling and are ligands for transmembrane receptors-CB1 and CB2 (cannabinoid receptor type 1 and 2) [11]. Activation of CB1 is responsible for ROS generation, whereas CB2 prevents ROS generation and inflammation, while also stimulating the MAP kinase (mitogen-activated protein kinase) pathway [12].

In connection with the permanent exposure to harmful UV radiation, cells from different skin layers-epidermal keratinocytes and dermal fibroblasts create a number of defense mechanisms that protect against UV-induced oxidative stress. Examples of such mechanisms are the high activity of repair and antioxidant enzymes, a large pool of non-enzymatic antioxidants, and redox-sensitive transcription factors including Nrf2 that is responsible for cytoprotective protein expression [13].

Despite the above well-developed mechanisms, skin cells exposed to long-lasting UV radiation are vulnerable to the depletion of the natural antioxidants present and therefore there is still a need for compounds with cytoprotective properties against UV-induced changes. One potential source are seed oils, the main source of phospholipids and triacylglycerols, which are solvents for other lipophilic compounds, i.e., sterols, fat-soluble vitamins (vitamin A and E), carotenoids, phenolic compounds, and free fatty acids [14]. One good source of seed oil is sea buckthorn (*Hippophae rhamnoides* L.) [15], which is increasingly appearing in skin care preparations. Its antioxidant potential is based on high content of polyphenols, vitamins (C and E), carotenoids as well as sterols [16,17,18]. In addition to the antioxidant activity, sea buckthorn also shows antibacterial and antifungal activity [19]. Carotenoids contained in the oil stimulate collagen synthesis, while phytosterols regulate inflammatory processes and have anticancer effects [20]. Because of the high concentration of these compounds sea buckthorn seed oil has been found as a promising therapeutic agent in the treatment of dermatitis [21]. However, there is no research on the effect of sea buckthorn seed oil on the UV-induced oxidative lipid metabolism of skin cells.

The aim of this study was to estimate the effect of sea buckthorn seed oil on the correlation between redox balance, lipid metabolism and endocannabinoid system in in vitro cultured human keratinocytes and fibroblasts exposed to the UV-radiation.

## 2. Materials and Methods

### 2.1. Examination of Sea Buckthorn Seed Oil Composition

Sea buckthorn (*Hippophae rhamnoides* L.) seed oil fatty acids levels as well as vitamins A and E were described below in the paragraphs Determination of fatty acids levels and Determination of non-enzymatic antioxidant levels.

#### 2.1.1. Determination of Squalene Levels

Squalene level was analyzed with separation on HP-5ms capillary column (0.25 mm; 0.25 µm, 30 m) of GC/MS system (Agilent Technologies, Palo Alto, CA, USA) with 7890A GC–7000 quadrupole MS/MS (Agilent Technologies, Palo Alto, CA, USA). Operating conditions were as follows: Oven programming −50 °C (10 min), rate 2 °C/min to 310 °C (30 min); ion source (EI) −230 °C; electron energy −70 eV [22]. The mass spectrometer source was run in selective ion monitoring (SIM) mode for the following ions: 191 and 81 *m*/*z*.

#### 2.1.2. Phytosterols Profile

The content of sterols in oils was determined by GC/MS system with 7890A GC–7000 quadrupole MS/MS (Agilent Technologies, Palo Alto, CA, USA) [23]. Samples were separated on HP-5ms capillary column (0.25 mm; 0.25 µm, 30 m). Operating conditions were as follows: oven programming −50 °C (2 min), rate 15 °C/min to 230 °C, to 310 °C at the rate of 3 °C/min (10 min); ion source (EI) −230 °C; electron energy −70 eV. The mass spectrometer source was run in selective ion monitoring (SIM) mode for the following ions: 372 and 217 *m*/*z* for 5-α-cholestane (IS), 458 and 368 *m*/*z* for cholesterol, 470 and 255 *m*/*z* for brassicasterol, 382 and 343 *m*/*z* for campesterol, 394 and 255 *m*/*z* for stigmasterol and 396 and 357 *m*/*z* for β-sitosterol. The quantifications were carried out using the internal standard method.

#### 2.1.3. Determination of β-Carotene Levels

HPLC methods was used to detect the level β-carotene [24]. Samples were dissolved in 1 mL of 2-propanol and 20 μL was taken analysis. Separation was performed using C18 column (150 nm × 4.6 mm) with UV detection at 454 nm. The concentration was determined using a calibration curve range 0.5–50 mg/L.

### 2.2. Cell Culture

Human skin cell lines used in experiment were obtained from American Type Culture Collection and cultured cultured in a humidified atmosphere of 5% CO_2_ at 37 °C. Human immortalized keratinocytes (CDD 1102 KERTr) were transformed with human papillomavirus 16 (HPV-16) E6/E7, while fibroblasts (CCD 1112Sk) were isolated from normal foreskin of Caucasian newborn male and used in passage 11. The growth media for each line were prepared as follows: for keratinocytes-keratinocyte-SFM medium with 1% Bovine Pituitary Extract (BPE) and human recombinant Epidermal Growth Factor (hEGF); for fibroblasts-Dulbecco’s Modified Eagle Medium (DMEM) with 10% fetal bovine serum (FBS). Media were supplemented with 50 U/mL penicillin and 50 μg/mL streptomycin. All sterile and cell culture reagents were obtained from Gibco (Grand Island, NY, USA).

Cells were exposed to UV radiation after reaching the 70% confluence. Radiation doses for keratinocytes were 30 J/cm^2^ for UVA (365 nm, power density at 4.2 mW/cm^2^) and 60 mJ/cm^2^ for UVB (312 nm, power density at 4.08 mW/cm^2^), while for fibroblasts were 20 J/cm^2^ for UVA and 200 mJ/cm^2^ for UVB (Bio-Link Crosslinker BLX 312/365, Vilber Lourmat, Germany). The distance of the cells from lamps was 15 cm. Exposure doses were chosen corresponding to 70% cell viability measured by the MTT (3-(4,5-dimethylthiazol-2-yl)-2,5-diphenyltetrazolium bromide) assay [25], what was previously shown as a doses that lead to the activation of pro-oxidative conditions, response of cell antioxidants, as well as activation of Nrf2 pathway and increases endocannabinoid system action [6]. Control cells were incubated in parallel without irradiation.

To examine the effect of sea buckthorn (*Hippophae rhamnoides* L.) seed oil (produced by “Szarłat” M i W Lenkiewicz Sp. J., Poland) on UV radiated skin cells, after keratinocytes and fibroblasts were exposed to UV radiation they were incubated 24 h under standard conditions in medium containing 500 ng/mL plant oil in 0.1% DMSO. The oil concentration was chosen corresponding to 100% cell viability compared to control cells measured by the MTT assay [25]. Parallel cells were cultured in medium containing plant oil without irradiation.

### 2.3. Examination of Pro-Oxidative Activity 

#### 2.3.1. Determination of ROS Generation

The superoxide anions generation was detected using stable nitroxide CM-radicals formation and detection with EPR spectrometer (Noxygen GmbH/Bruker Biospin GmbH, Germany) [10,26]. The results were reported in micromolar concentration of ROS per minute and normalised per milligram of protein.

#### 2.3.2. Determination of Pro-Oxidants Enzyme Activities

NADPH oxidase (NOX-EC 1.6.3.1) activity was analysed by luminescence measurement using lucigenin as luminophore [27]. Enzyme specific activity was described in relative luminescence units (RLU) per milligram protein.

Xanthine oxidase (XO-EC1.17.3.2) activity was estimated by uric acid formation from xanthine [28]. Enzyme specific activity was described in microunits per milligram of protein.

### 2.4. Examination of Antioxidant Defence System

#### 2.4.1. Determination of Antioxidant Enzymes Activity

Glutathione peroxidase (GSH-Px-EC.1.11.1.6) activity was assessed spectro-photometrically [29]. Enzyme specific activity was described in microunits per milligram of protein.

Glutathione reductase (GSSG-R-EC.1.6.4.2) activity was measured by monitoring the oxidation of NADPH at 340 nm [30]. Enzyme specific activity was described in milliunits per milligram of protein.

Superoxide dismutase (Cu/Zn–SOD-EC.1.15.1.1) activity was determined according to the method of Misra and Fridovich [31] as modified by Sykes [32]. Enzyme specific activity was calculated in milliunits per milligram of protein.

The thioredoxin reductase (TrxR-EC. 1.8.1.9) activity was measured using a commercially available kit (Sigma-Aldrich, St. Louis, MO, USA) in accordance with the included instruction [33]. Enzyme activity was measured in units of microunits per milligram of protein.

#### 2.4.2. Determination of Non-Enzymatic Antioxidant Levels

Thioredoxin level were quantified using the ELISA (enzyme-linked immunosorbent assay) [34]. The ELISA plates coated with samples were incubated at 4 °C overnight with anti-thioredoxin primary antibody per well (diluted in 1% bovine serum albumin in phosphate buffered saline (PBS)) (Abcam, Cambridge, MA, USA). After washing and blocking the plates were incubated with goat anti-rabbit secondary antibody solution (Dako, Carpinteria, CA, USA). As a chromogen substrate solution 0.1 mg/mL 3,3′, 5,5′-tetramethylbenzidine was used. Spectral absorption was read at 450 nm with the reference filter at 620 nm. Thioredoxin levels were described in micrograms per milligram of protein.

Glutathione level was measured using the capillary electrophoresis (CE) [35] with separation on 47 cm capillary operated at 27 kV. UV detection was set at 200 ± 10 nm. Calibration curve range of 1–120 nmol/mL (r^2^ = 0.9985) was prepared to determined GSH concentration that was subsequently normalized for milligrams of protein.

To detect the level of vitamins A and E HPLC method was used [36]. Vitamins were extracted using hexane. The concentration of vitamins detected at 294 nm was determined using a calibration curves range: 0.125–1 mg/L (r^2^ = 0.9998) for vitamin A, and 5–25 mg/L (r^2^ = 0.9999) for vitamin E.

#### 2.4.3. Determination of Protein Expression

Protein expression level was analyzed using Western blot technique [37]. Cell lysates or nuclear fractions (in the case of phosphorylated protein Nrf2) were separated by 10% Tris-Glycine SDS-PAGE (sodium dodecyl sulfate polyacrylamide gel electrophoresis). Following electrophoresis, separated proteins were transferred into a membrane and blocked with 5% skim milk. Primary antibodies against phospho-Nrf2 (pSer40), HO-1, Keap1, p21, p62, and peroxisome proliferator-activated receptors (PPARα, γ, and δ) (Sigma-Aldrich, St. Louis, MO, USA) were diluted 1:1000. Protein bands were visualized calorimetrically using the BCIP/NBT (5-bromo-4-chloro-3-indolyl-phosphate/nitro blue tetrazolium) Liquid substrate system (Sigma-Aldrich, St. Louis, MO, USA). Protein level was expressed as a percentage of control cells.

### 2.5. Examination of Lipid Metabolism

#### 2.5.1. Determination of Cellular Membrane Integrity by LDH Test

Keratinocyte and fibroblast cellular membrane integrity was monitored using the assay based on the determination of the release of lactate dehydrogenase (LDH) into the medium. Cells were seeded into a 96 well plates in 200 μL of growth media at 5 × 10^3^ for 24 h and then subjected to UV radiation or oil treatment range 5 ng/mL–5 mg/mL. Following 24 h incubation the activity of LDH in medium and in cell lysates was measured after 24 h. Spectrophotometric detection was carried out at 340 nm. The final concentrations of NADH and pyruvate in the reaction mixture were 1 mM and 2 mM, respectively [25]. The rate of LDH released from the treated cells was calculated by comparing its activity in medium to cell lysates. The results were expressed as the percentage of LDH activity obtained for control cells.

#### 2.5.2. Determination of Fatty Acids Levels

Fatty acids profiles were determined by gas chromatography [38]. Free fatty acids (FFA) and phospholipids (PL) were methylated to fatty acid methyl esters (FAMEs). FAMEs were analyzed by Clarus 500 Gas Chromatograph (Perkin Elmer, Shelton, CT, USA) following separation on capillary column (50 m × 0.25 mm, ID 0.2 μm, Varian, Walnut Creek, USA). Identification of FAMEs was made by comparison to their retention time with authentic standards. Quantitation was achieved using an internal standard method.

#### 2.5.3. Phospholipase A2 Activity

The activity of cPLA_2_ (cytosolic phospholipase A_2_) activity was measured using a commercially available kit (Cayman, No. 765021). To detect cPLA_2_ activity specific inhibitors of sPLA2 (thioetheramide-PC) and iPLA2 (bromoenol lactone) were added to samples [39]. Enzyme specific activity was presented in nanomol of free thiol released per minute normalized per milligram of protein.

#### 2.5.4. Determination of Lipid Peroxidation Products

Lipid peroxidation was estimated by measuring the level of 4-hydroxynonenal (4-HNE) and 8-Isoprostaglandin F2α.

Aldehydes were measured by GC/MS in selected ion monitoring (SIM) mode, as the O-PFB-oxime or O-PFB-oxime-TMS derivatives using benzaldehyde-D6 as an internal standard [40]. Aldehydes were analyzed using a 7890A GC—7000 quadrupole MS/MS (Agilent Technologies, Palo Alto, CA, USA) equipped with a HP-5ms capillary column (30-m length, 0.25-mm internal diameter, 0.25-µm film thickness). Target ion with a *m*/*z* 333.0 and 181.0 for 4-HNE-PFB-TMS and *m*/*z* 307.0 for IS derivatives were selected. Obtained results were normalized for milligrams of protein.

8-Isoprostaglandin F2α (8-isoPGF2α) level was measured using LC-MS (Agilent 1290 LC with triple quadruple mass spectrometer in negative multiple reaction mode [41]. SEP-PAK C18 column were used to samples purification. Target ions with a *m*/*z* 353→193 were selected. Target ion with a *m*/*z* 353→193 was selected.

#### 2.5.5. Determination of Endocannabinoid System

Anandamide (AEA) and 2-arachidonoylglycerol (2-AG) were quantified using UPLC-MS/MS system (Agilent 1290 UPLC system interfaced with an Agilent 6460 triple quadrupole mass spectrometer) [42]. As internal standards AEA-d8 and 2-AG-d8 were used. The samples were analyzed in positive-ion mode using multiple reaction monitoring (MRM) and the transition of the precursor to the product ion were: *m*/*z* 348.3→62.1 for AEA, and *m*/*z* 379.3→287.2 for 2-AG. Endocannabinoids concentrations were expressed as femtomols per milligram of protein.

The levels of endocannabinoid receptors CB1/2 were measured by Western blotting described above (*Determination of protein expression)*. Primary monoclonal antibodies against CB1 and CB2 (Santa Cruse Biotechnology, Santa Cruz, CA, USA) were used at a concentration of 1:1000. Receptor level was expressed as a percentage of control cells.

### 2.6. Statistical Analysis

Data were analyzed using standard statistical analyses, including *t*-test, and the results are expressed as the mean ± standard deviation (SD) for *n* = 5. Experiment was performed on cell lines and all replicates were technical repetitions and do not reflect biodiversity. *p*-values less than 0.05 were considered significant.

## 3. Results

### 3.1. The Sea Buckthorn Seed Oil Composition

The composition of sea buckthorn seed oil is presented in Table 1. In addition to the large amount of fatty acids in the free form as well as in the form of DG, TAG and PL, the seed oil of sea buckthorn also contains phytosterols, such as cholesterol, brassicrakol, campesterol, stigmasterol and β-sitosterol, as well as antioxidants such as vitamins A and E, squalene and β-carotene.

### 3.2. The Effect of Sea Buckthorn Seed Oil on Skin Cells Redox Balance

Sea buckthorn seed oil treatment was found as a factor that partially prevented the UV-induced disturbances in redox balance occurring in experimental skin cells. It was found that fibroblast incubation with sea buckthorn oil caused decrease in ROS generation by approximately 25% as well as a decrease in the activity of NOX and XO by approximately 15% following UVA and 25% following UVB radiation. Similar character of changes was observed in the case of keratinocytes only following UVB radiation, were sea buckthorn oil caused decrease in NOX and XO activity by 10% and 15%, respectively (Table 2).

Simultaneously, sea buckthorn oil altered the antioxidant system (Table 3), what was primarily visible in the case of the non-enzymatic antioxidant thioredoxin, its level was significantly increased by sea buckthorn oil in the keratinocytes and fibroblasts exposed to UV radiation by about 60% and 37% respectively in the case of UVA, and by 37% and 175%, respectively in the case of UVB radiation. Sea buckthorn oil also enhanced the level of glutathione in UV exposed cells; it was increased 2.5 fold in the UV irradiated keratinocytes, and a 25% and 80% increase was observed in the case of UVA and UVB irradiated fibroblasts, respectively. In the case of vitamin levels, sea buckthorn oil treatment caused an increase in vitamin A levels in UVA and UVB irradiated keratinocytes by approximately 50% and 60%, respectively; and in UVA and UVB irradiated fibroblasts by about 20% and 15%, respectively. Also, the level of vitamin E following UV radiation was partially restored; by 55% in keratinocytes and by 30% in fibroblasts following both UV types. Described changes were accompanied by enhanced GSH-Px activity in keratinocytes (by 15% following UVA and even 2 times following UVB radiation). Moreover, sea buckthorn oil cell treatment lead to 4- and 3-fold increase in TrxR activity in keratinocytes following UVA and UVB radiation, and by 20% and 30% increases in the enzyme activity in fibroblasts following UVA and UVB radiation, respectively. However, in the case of SOD activity sea buckthorn oil additionally decreased its activity by 15% in the case of both cell lines.

### 3.3. The Effect of Sea Buckthorn Seed Oil on Skin Cells Transcription Factors

**Figure 1:**
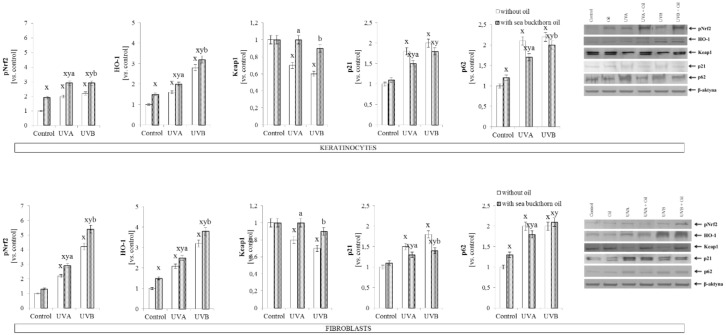
The expression of transcription factor phospho-Nrf2 (pSer40), its main target HO-1, and its inhibitor/activators-Keap1, p21, p62 in keratinocytes and fibroblasts after exposure to UVA (30 J/cm^2^ and 20 J/cm^2^) and UVB radiation (60 mJ/cm^2^ and 200 mJ/cm^2^, respectively) and sea buckthorn seeds oil (500 ng/mL) treatment. Mean values ± SD of five independent experiments are presented. ^**x**^ statistically significant differences vs. control group, *p* < 0.05; **^y^** statistically significant differences between UVA/UVB and oil treated groups vs. oil treated control group, *p* < 0.05; **^a^** statistically significant differences between UVA and oil treated group vs. UVA treated group, *p* < 0.05; **^b^** statistically significant differences between UVB and oil treated group vs. UVB treated group, *p* < 0.05.

Sea buckthorn oil treatment also stimulated the antioxidant system, increased expression of phosphorylated factor Nrf2 in skin cells following UV radiation was observed in both cell lines as well as in the case of expression of Nrf2 targeted protein—HO-1. On the other hand, sea buckthorn oil restored the decreased activity following UV radiation, Keap1 level in UVA irradiated skin cells, and in UVB irradiated keratinocytes. Simultaneously, sea buckthorn oil lead to the decrease in Nrf2 activators—p21 and p62, in skin cells following both UVA and UVB radiation (Figure 1).

**Figure 2:**
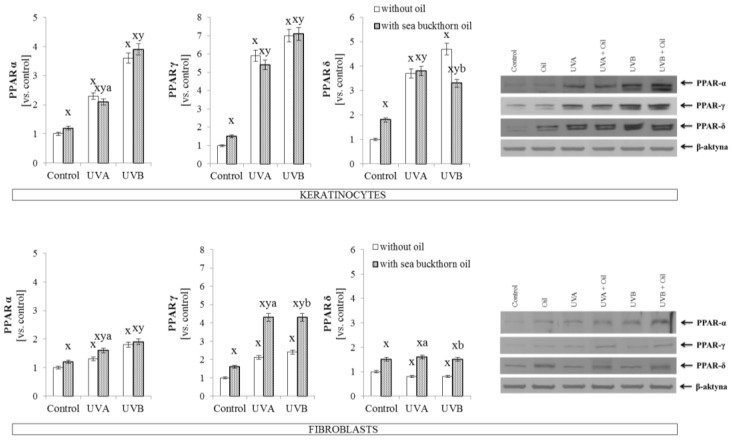
The expression of peroxisome proliferator-activated receptors (PPARα, γ, and δ) in keratinocytes and fibroblasts after exposure to UVA (30 J/cm^2^ and 20 J/cm^2^) and UVB radiation (60 mJ/cm^2^ and 200 mJ/cm^2^, respectively) and sea buckthorn seeds oil (500 ng/mL) treatment. Mean values ± SD of five independent experiments are presented. ^**x**^ statistically significant differences vs. control group, *p* < 0.05; **^y^** statistically significant differences between UVA/UVB and oil treated groups vs. oil treated control group, *p* < 0.05; **^a^** statistically significant differences between UVA and oil treated group vs. UVA treated group, *p* < 0.05; **^b^** statistically significant differences between UVB and oil treated group vs. UVB treated group, *p* < 0.05.

Additionally, sea buckthorn oil treatment following UV radiation induced various effects in keratinocytes and fibroblasts and in the other transcription factors such as peroxisome proliferator-activated receptors (PPARα, γ, and δ). In keratinocytes sea buckthorn oil lead to a decrease by 8% in PPARα expression following UVA radiation and by 20% in PPARδ expression following UVB radiation, while in UVA and UVB irradiated fibroblasts increases by 10% in PPARα, by 50% in PPARδ, and 2-fold in PPARγ expression were observed following sea buckthorn oil treatment (Figure 2).

### 3.4. The Effect of Sea Buckthorn Seed Oil on Skin Cells Lipid Metabolism

**Figure 3:**
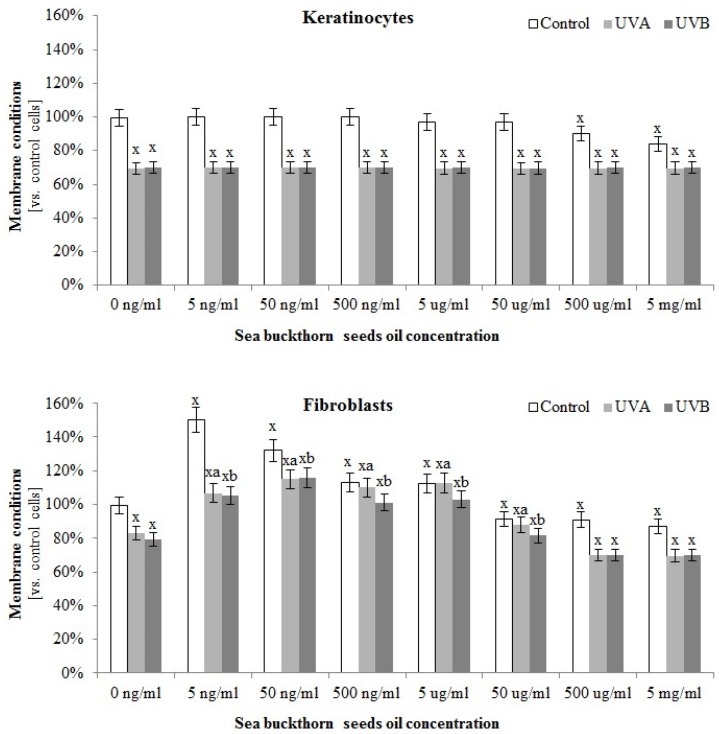
The effect of different concentrations ranging 50 ng/mL–5 µg/mL of sea buckthorn seed oil on the membrane conditions of control and UV irradiated keratinocytes and fibroblasts measured by LDH test. Total doses of UV irradiation for each cell line were: 30 J/cm^2^ and 60 mJ/cm^2^ for keratinocytes, and 20 J/cm^2^ and 200 mJ/cm^2^ for fibroblasts for UVA and UVB, respectively. Mean values ± SD of five independent experiments are presented. ^**x**^ statistically significant differences vs. control group, *p* < 0.05; **^y^** statistically significant differences between UVA/UVB and oil treated groups vs. oil treated control group, *p* < 0.05; **^a^** statistically significant differences between UVA and oil treated group vs. UVA treated group, *p* < 0.05; **^b^** statistically significant differences between UVB and oil treated group vs. UVB treated group, *p* < 0.05.

The composition of sea buckthorn oil also had an impact on skin cells lipid metabolism. The greatest protective effect of this oil on membrane conditions was observed in the case of fibroblasts, where sea buckthorn oil not only reduced the permeability of the membrane compared to the control cells measured by LDH test (in the concentration rage 5 ng/mL–5 µg/mL), but also significantly prevented UVA and UVB-induced membrane damage (in the concentration rage 5 ng/mL–50 µg/mL). These effects were not observed in the case of keratinocytes (Figure 3).

On the other hand, sea buckthorn oil treatment on skin cells significantly increased the level of all detected phospholipids as well as free fatty acids in both keratinocytes and fibroblasts (Table 4). Moreover, sea buckthorn oil influenced the UV-decreased level of numerous fatty acids, which was particularly evident in the case of keratinocytes and fibroblasts treated with oil following UVB radiation. Additionally, sea buckthorn oil treatment effects on short fatty acids (16:0; 16:1) in UVA irradiated cells was stronger in fibroblasts than in keratinocytes, while sea buckthorn oil treatment effect on the level of longer fatty acids (20:4) was visible only in the case of keratinocytes (Table 3). The Figure S1 presents the correlation between the level of free fatty acids in sea buckthorn seeds oil and changes in the level of free fatty acids in keratinocytes and fibroblasts.

**Figure 4:**
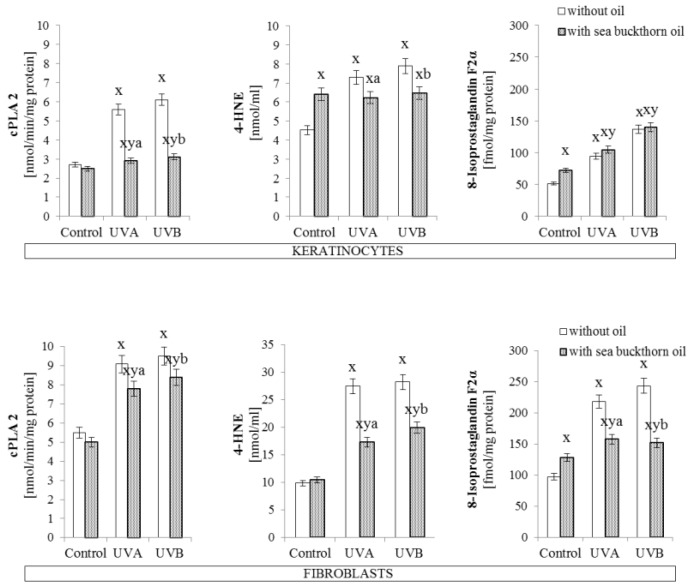
The activity of phospholipase A2 and level of lipid peroxidation products (4-HNE and 8-Isoprostaglandin F2α) in keratinocytes and fibroblasts after exposure to UVA (30 J/cm^2^ and 20 J/cm^2^) and UVB radiation (60 mJ/cm^2^ and 200 mJ/cm^2^, respectively) and sea buckthorn seeds oil (500 ng/mL) treatment. Mean values ± SD of five independent experiments are presented. ^**x**^ statistically significant differences vs. control group, *p* < 0.05; **^y^** statistically significant differences between UVA/UVB and oil treated groups vs. oil treated control group, *p* < 0.05; **^a^** statistically significant differences between UVA and oil treated group vs. UVA treated group, *p* < 0.05; **^b^** statistically significant differences between UVB and oil treated group vs. UVB treated group, *p* < 0.05.

Obtained results showed that sea buckthorn oil partially prevented UV-induced increase in phospholipase A2 activity by 50% in the case of UV irradiated keratinocytes, and by about 15% in the case of UV irradiated fibroblasts (Figure 4). As a result of UV-induced lipid oxidative metabolism a strong increase in the level of lipid peroxidation products was observed. Sea buckthorn oil treatment significantly prevented these changes in the case of 4-HNE levels, which was decreased by 15% in oil treated keratinocytes, and 30% in oil treated fibroblasts. Also, the level of isoprostaglandin in fibroblasts exposed to UV radiation was decreased by 25% following sea buckthorn oil treatment. However, in the case of isoprostaglandin levels in keratinocytes sea buckthorn oil lead to increases by approximately 20% under standard conditions and did not have a significant effect on irradiated cells (Figure 4).

**Figure 5:**
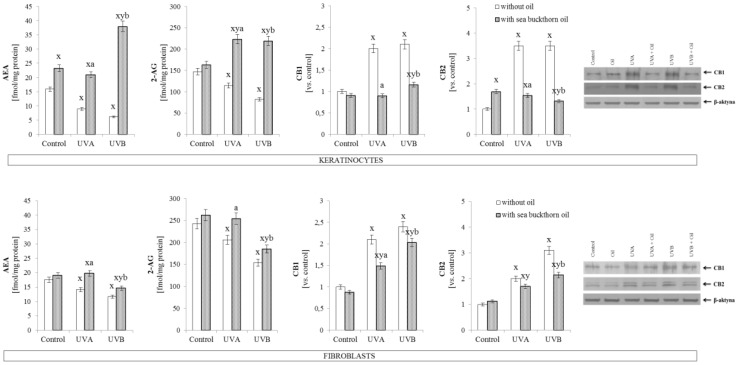
The level of endocannabinoids (AEA, 2-AG) and endocannabinoid receptors (CB1, CB2) in keratinocytes and fibroblasts after exposure to UVA (30 J/cm^2^ and 20 J/cm^2^) and UVB radiation (60 mJ/cm^2^ and 200 mJ/cm^2^, respectively) and sea buckthorn seeds oil (500 ng/mL) treatment. Mean values ± SD of five independent experiments are presented. ^**x**^ statistically significant differences vs. control group, *p* < 0.05; **^y^** statistically significant differences between UVA/UVB and oil treated groups vs. oil treated control group, *p* < 0.05; **^a^** statistically significant differences between UVA and oil treated group vs. UVA treated group, *p* < 0.05; **^b^** statistically significant differences between UVB and oil treated group vs. UVB treated group, *p* < 0.05.

Sea buckthorn oil treatment on UV irradiated skin cells was found as a factor influencing the endocannabinoid system. In both cell lines a significant increase in endocannabinoid levels were observed, however, keratinocytes exhibited greater susceptibility to oil treatment (approximately 3-fold and 7-fold increase in the case of AEA; and 2-fold and 3-fold increase in the case of 2-AG, compared to UVA and UVB irradiated cells, respectively, were observed). Moreover, endocannabinoid receptor expression was decreased by half in relation to UV irradiated keratinocytes. Parallel, in fibroblasts sea buckthorn oil following UV irradiation caused increases in the endocannabinoid levels by 25–30% compared to UVA or UVB irradiated cells. Also, the expression of endocannabinoid receptor CB1 was decreased by 25% compared to UVA or UVB irradiated cells. However, in the case of receptor CB2, sea buckthorn oil treatment decreased its expression only in fibroblasts exposed to UVB radiation (Figure 5).

## 4. Discussion

Since the major components of the outer skin layer are lipids, it can be assumed that the use of lipid derivatives will enhance skin conditions. Therefore, plant oils can be used as a source of compounds that may protect skin cells due to their multidirectional activities, including anti-inflammatory, antibacterial and antioxidants, as well as the source of fatty acids [43]. Sea buckthorn (*Hippophae rhamnoides*) seed oil has been traditionally used for accelerating wound healing [44] as well as treating skin diseases such as the first stage of atopic dermatitis [45], however it is not widely used in skin protection [46].

### 4.1. Sea Buckthorn Seeds Oil Effect on Antioxidant System

Due to the high content of polyphenolic compounds and vitamins, the sea buckthorn extract has been found as a antimicrobial factor, with strong antioxidant activity in in vitro examinations [47]. Moreover, it has been suggested that sea buckthorn seed oil may reduce ROS levels under oxidative conditions by its ability to scavenge free radicals [48].

This study indicates the effectiveness of the antioxidant actions of sea buckthorn oil in relation to the oxidative conditions found in skin cells exposed to UV radiation. However, in physiological conditions it is not so unambiguous, because by increasing the activity of cytosolic enzymes such as NADPH oxidase and xanthine oxidase this oil contributes to the intensification of ROS generation. After UV radiation, sea buckthorn oil promotes reduction of the above enzyme activity and consequently ROS generation in fibroblasts. This may be the result of a stronger fibroblast susceptibility to external factors including oxidative factors such as UVA and UVB radiation and for antioxidative factors in oils, which was also indicated for pomegranate seed oil [49]. It has been suggested that high content of unsaturated acids in plant oils are substrates for cellular oxygenases which can be a source of oxidation products in skin cells [50].

Independent of modifications of prooxidative conditions, sea buckthorn oil promotes increases in antioxidant enzyme activity and non-enzymatic antioxidant levels in control cells as well as cells treated with UV irradiation. Results of this study partially confirm earlier observations that sea buckthorn oil may lead to enhanced antioxidant abilities by promotion of glutathione accumulation in the whole animal body in the case of rats treated with this oil [51]. It was also shown that supplementation of rats with compounds of sea buckthorn extracts activate the enzymes superoxide dismutase, catalase, glutathione peroxidase, glutathione reductase and glutathione S-transferase in animal blood [52]. However, in this study sea buckthorn oil shows the tendency to decrease fibroblast Cu, Zn-SOD activity in control and UV-irradiated cells. This may be explained by the sea buckthorn oils ability to capture electrons and extinguish the singlet oxygen or superoxide anion [53] that is the main substrate of SOD in cell cytoplasm. Another important cellular redox regulator is the thioredoxin system consisting of thioredoxin and thioredoxin reductase (TrxR) [54]. This expression is diminished in skin cells after UV irradiation and enhanced after sea buckthorn oil treatment of keratinocytes and fibroblasts. Previously it has been shown that this system plays a significant role in the pathogenesis of a number of diseases, but it also participates in cellular protection against toxic compounds, which are reduced by thioredoxin or inhibited by thioredoxin reductase [55,56]. It has been indicated that sea buckthorn includes high levels of proteins containing thioredoxin domains and prevents the disulfide bond formation in oxidizing environments and stabilizing the tertiary and quaternary structure of proteins [57]. It has also been shown that Trx and TrxR synthesis is induced after exposure to prooxidative factors [58], this explains the strong positive response of this system to UV irradiation, particularly in fibroblasts. Therefore, enhanced effectiveness of this system after oil treatment may explain reduction of oxidative stress in these cells.

Cellular antioxidant responses dependent on proteins is promoted by the transcription factor Nrf2 which is responsible for cytoprotective gene transcription [59]. Under physiological conditions, cytoplasmic Nrf2 is bound to Keap1 [60], but oxidative stress caused by UV radiation was found to diminish Keap1 expression which prevents Nrf2–Keap1–Cul3 complex formation. Moreover, as observed in this paper, high levels of ROS and electrophiles such as 4-HNE may lead to the oxidation of Keap1 cysteine residues causing lack of binding and/or dissociation of Nrf2 from the complex resulting in its translocation into the nucleus, which was reported earlier [61]. Nrf2 activation, demonstrated also as its targeted genes-HO-1 expression, leads to cellular protection against pro-oxidative conditions. Sea buckthorn oil treatment of UV irradiated skin cells acts in two ways—it supports the cellular antioxidant capacity by the activation of Nrf2, and at the same time, affects the expression of proteins strongly related with Nrf2 activation. The results showing enhanced phospho-Nrf2 translocation to the nucleus despite of the increased Keap1 level in cytoplasm clearly suggest that sea buckthorn oil disrupts the Nrf2–Keap1 complex formation. Moreover, sea buckthorn seed oil decreases UV-induced levels of p21 and p62, reducing the possibility of these proteins to create adducts with Nrf2 or Keap1, thus encouraging Nrf2 inhibition [59,62]. Keap1 might also create adducts with small antioxidant molecules such as melatonin or GSH, what also prevents Nrf2-Keap1 binding, however, it does not lead to Keap1 degradation [63,64]. Melatonin has been previously shown to be downregulated in skin cells following UV radiation [65,66], while UV-induced decrease in GSH level, what is shown in this study is significantly enhanced as a result of sea buckthorn oil treatment. These modifications to the activators and inhibitors of the Nrf2 pathway are not able to cancel the protective effect of sea buckthorn oil to counteract prooxidative conditions.

### 4.2. Sea Buckthorn Seeds Oil Effect on Lipid Metabolism

The effects of sea buckthorn seed oil on redox balance can directly protect the metabolism of skin cells. Maintaining redox homeostasis is important for the physiological metabolism of lipids, which is important for the proper functioning of cells. Phospholipid protection may be associated with beneficial fatty acids present in sea buckthorn oil [67], which influences the skin cells fatty acid profile. The strongest correlation between oil fatty acids and increase in the cells fatty acid level is visible in the case of free fatty acids (FFA) regardless of whether that were control cells or treated with UV radiation. This relationship between FFA composition of the applied oil and cells might be associated with 24 h incubation with oil when cells were able only to take up and assimilate FFA from fatty rich oil composition. Sea buckthorn oil is rich in palmitoleic acid (16:1), which has been reported to play a role in many metabolic processes including intracellular lipid mediated signal transduction [68,69], which includes lipid metabolism, and is also responsible for maintaining the fluidity of biological membranes [70,71,72]. Palmitoleic acid levels are enhanced in the phospholipid profile of keratinocytes and fibroblasts after sea buckthorn oil treatment, this also causes the reduction of mRNA expression of proinflammatory genes, i.e., TNF-α [73], therefore this oil may affect intracellular signaling based on PI3K/Akt kinase cascades, and have anti-inflammatory activity, which was also confirmed by experiments on rats with diabetes [74]. Moreover, sea buckthorn oil contains a large amount of linoleic and α-linolenic acids, which can enhance the level of phospholipid α-linolenic acid in cells [75,76]. α-Linolenic acid contained in sea buckthorn oil has been found also as a source of eicosanoid prostaglandin E1, a signal precursor that produces antibacterial and cytoprotective extracellular fluids [77]. Shown in this study sea buckthorn oil added to skin cells enhances the level of these acids in phospholipid and free PUFAs fractions. Skin cells phospholipid PUFAs fraction is also enriched in γ-linolenic acid that is a precursor of anti-inflammatory eicosanoids, such as the 1-series prostaglandins and 15-hydroxyeicosatrienoic acid (15-HETrE) [78].

It has been previously shown that UV radiation significantly disturbs the metabolism of skin cell membrane phospholipids [6]. In this study, active compounds contained in sea buckthorn oil enhances the cell’s antioxidant abilities and prevents lipid peroxidation [47] as well as enzymatic phospholipid metabolism. Sea buckthorn oil enhances, both phospholipid and free PUFA levels in keratinocytes and fibroblasts, as well as increases the ROS level favored by ROS-dependent lipid peroxidation manifested as oxidative fragmentation with enhanced 4-HNE levels and as oxidative cyclisation with enhanced 8-isoprostane levels. Independently of that, one of the main sources of peroxidation products-arachidonic acid (AA) is most enhanced in PUFAs of both skin cell lines, what indicates for huge tributary of this acid from used oil. As a result of AA peroxidation, increases in the 4-HNE levels can directly act as a signaling molecule or through protein adduct formation which significantly influences their structure and activity [79]. Phospholipid AA is also metabolized by enzymes among which the most important are phospholipases including PLA2 [80]. It has been previously shown that UV radiation significantly increases PLA2 activity [9,81], while PLA2 inhibition improves skin conditions [82]. Sea buckthorn oil reveals similar as other plant oils PLA2 inhibition properties, preventing UV-induced lipid metabolism [83]. Despite the observed decrease in PLA2 activity, the levels of endocannabinoids are increased after using sea buckthorn oil. It is believed that anandamide is a partial or full agonist of the CB1 receptor, depending on tissue and conditions, and is suggested to have low efficacy for CB2 receptors, whereas 2-AG is a full agonist of both CB1 and CB2 receptors [84]. However, an elevated level of endocannabinoids is accompanied by a down-regulation of cannabinoid receptors. Such response may indicate that redox and inflammatory regulation is independent from the cannabinoid receptors. Moreover, oil-induced changes in endocannabinoids level may influence the skin neuroendocrine capabilities regulated by UV radiation [85], what leads to disorders in steroid hormones, neuropeptides, and neurotransmitters biosynthesis [86]. Regardless of the above, it has been shown that a high level of AA delivered to skin cells from sea buckthorn oil may result in increased generation of the 4-series leukotrienes, which have a strong pro-inflammatory and hyperproliferative effect [87].

Endocannabinoid levels are enhanced by treating skin cells with sea buckthorn oil, which are agonists of peroxisome proliferator-activated receptors (PPAR). It is known that PPARs are activated by fatty acids and their derivatives, including lipid peroxidation products like 4-HNE, which act as PPAR-α agonists [88,89]. PPARs act as modulators of cellular processes including lipid metabolism, and thus create a lipid signaling network between the cell surface and the nucleus [89]. Enhanced expression of fibroblast PPARs indicates that sea buckthorn seed oil has anti-inflammatory activity. PPAR-α controls the expression of proteins that participate in inflammatory response [89], therefore enhanced activation of PPAR-α observed in fibroblasts indicates preventing NF-κB-dependent inflammation [90]. It has also been shown that anandamide as a ligand of PPAR-α can participate in its anti-inflammatory effect through impaired production of TNF-α [91]. Similarly, enhanced expression of PPAR-γ decreases the expression of TNF-α [92]. Moreover, sea buckthorn ethanolic extract has been beneficial in reducing fat pad mass and preventing weight gain in mice. The extract was effective in producing hypoglycemic effects in animals through up-regulating PPAR-γ and PPAR-α gene expression [92].

## 5. Conclusions

In summary, sea buckthorn oil significantly stimulates the antioxidant system in keratinocytes and fibroblasts. Therefore, sea-buckthorn seed oil prevents UV-induced impair in redox systems as well as lipid metabolism disorders in skin fibroblasts and keratinocytes, which makes it a promising natural substance in skin photo-protection. However, the influence of UV radiation on the stability and durability of oil components, their interactions and impact on the metabolism of skin cells has not been studied, therefore it is believed that current studies do not allow recommending the use of sea buckthorn seed oil in direct exposure to UV radiation.

## Figures and Tables

**Table 1:** The fatty acids composition, antioxidant components and phytosterols in sea buckthorn (*Hippophae rhamnoides* L.) seeds oil. Mean values ± SD of five independent experiments are presented.

**Fatty Acids Composition of Sea Buckthorn Needs Oil**					
	**PL**	**TG**	**DG**	**FFA**	
	**mg/mL**				
14:0	0.060 ± 0.003	4.58 ± 0.14	0.240 ± 0.007	0.070 ± 0.003	
16:0	1.14 ± 0.03	308.60 ± 9.25	3.10 ± 0.09	1.65 ± 0.05	
18:0	0.51 ± 0.02	8.05 ± 0.24	0.47 ± 0.01	0.100 ± 0.003	
20:0	n.d.	2.13 ± 0.06	0.130 ± 0.003	0.060 ± 0.002	
22:0	n.d.	0.95 ± 0.03	0.140 ± 0.003	n.d.	
24:0	n.d.	1.08 ± 0.03	0.170 ± 0.004	0.070 ± 0.002	
SFA	1.71 ± 0.05	325.40 ± 9.76	4.26 ± 0.13	1.95 ± 0.06	
16:1n7	0.48 ± 0.01	310.85 ± 9.33	4.56 ± 0.14	2.22 ± 0.07	
18:1n9c	0.150 ± 0.005	45.34 ± 1.36	0.89 ± 0.03	0.34 ± 0.01	
18:1n7	0.110 ± 0.003	45.25 ± 1.35	0.83 ± 0.02	0.48 ± 0.01	
18:2n6	0.230 ± 0.007	87.68 ± 2.63	7.15 ± 0.21	1.53 ± 0.05	
18:3γ	n.d.	1.79 ± 0.05	n.d.	n.d.	
18:3α	0.280 ± 0.008	7.05 ± 0.21	n.d.	0.210 ± 0.006	
MUFA	0.74 ± 0.02	401.44 ± 12.04	6.29 ± 0.19	3.04 ± 0.09	
PUFA	0.51 ± 0.02	96.51 ± 2.90	7.15 ± 0.21	1.74 ± 0.05	
USFA	1.25 ± 0.04	497.95 ± 14.94	13.44 ± 0.40	4.78 ± 0.14	
Sum	2.95 ± 0.09	823.35 ± 24.70	17.70 ± 0.53	6.73 ± 0.20	
**Antioxidant Components of Sea Buckthorn Needs Oil [mg/100 g]**					
Squalene	β-Carotene		Vitamin E	Vitamin A	
240±11	15.11 ± 0.46		98.92 ± 2.89	648.0 ± 19.7	
**Phytosterols in Sea Buckthorn Needs Oil [mg/100 g]**					
Cholesterol	Brassicasterol	Campesterol	Stigmasterol	β-Sitosterol	Total phytosterols
0.51 ± 0.01	4.90 ± 0.14	17.61 ± 0.48	4.95 ± 0.12	223.88 ± 6.76	251.87 ± 7.48

Abbreviations: PL: phospholipid; DG: diacylglycerol; TG: triacylglycerol; EFA: essential fatty acid; n.d.: not detected.

**Table 2:** The reactive oxygen species (ROS) generation, xanthine oxidase and NADPH oxidase activity in keratinocytes and fibroblasts after exposure to UVA (30 J/cm^2^ and 20 J/cm^2^) and UVB radiation (60 mJ/cm^2^ and 200 mJ/cm^2^, respectively) and sea buckthorn seeds oil (500 ng/mL) treatment.

Prooxidative Parameters		Keratinocytes			Fibroblasts		
	Oil	Control	UVA	UVB	Control	UVA	UVB
ROS [nM/min/mg protein]	-	32.8 ± 1.6	89.1 ± 4.3 ^x^	98.9 ± 4.8 ^x^	50.6 ±2.4	126.5 ± 6.1 ^x^	132.1 ± 6.4 ^x^
	+	63.5 ± 3.1 ^x^	80.8 ± 3.9 ^xy^	96.5 ± 4.7 ^xy^	54.7 ±2.6	94.6 ± 4.6 ^xya^	96.5 ± 4.7 ^xyb^
NOX [RLU/mg protein]	-	158 ± 7.7	229 ± 11.2 ^x^	256 ± 12.5 ^x^	179 ± 8.7	321 ± 15.7 ^x^	398 ± 19.5 ^x^
	+	173 ± 8.4	231 ± 11.3 ^xy^	227 ± 14.0 ^xyb^	216 ± 10.5 ^x^	268 ± 13.1 ^xya^	321 ± 15.7 ^xyb^
XO [mU/mg protein]	-	164 ± 8.1	267 ± 13.1 ^x^	297 ± 14.5 ^x^	114 ± 5.5	248 ± 12.1 ^x^	487 ± 23.8 ^x^
	+	158 ± 7.7	248 ± 12.1 ^xy^	254 ± 12.4 ^xyb^	278 ± 13.6 ^x^	215 ± 10.5 ^xya^	348 ± 17.1 ^xyb^

Mean values ± SD of five independent experiments are presented. ^**x**^ statistically significant differences vs. control group, *p* < 0.05; ^**y**^ statistically significant differences between UVA/UVB and oil treated groups vs. oil treated control group, *p* < 0.05; ^**a**^ statistically significant differences between UVA and oil treated group vs. UVA treated group, *p* < 0.05; **^b^** statistically significant differences between UVB and oil treated group vs. UVB treated group, *p* < 0.05. Abbreviations: UVA: ultraviolet type A; UVB: ultraviolet type B; ROS: reactive oxygen species; NOX: NADPH oxidase; XO: xanthine oxidase.

**Table 3:** The activity of antioxidant enzymes (GSH-Px, GSSG-R, SOD, TrxR) and the level of non-enzymatic antioxidants (GSH, Trx, vitamins A, E, and C) in keratinocytes and fibroblasts after exposure to UVA (30 J/cm^2^ and 20 J/cm^2^) and UVB radiation (60 mJ/cm^2^ and 200 mJ/cm^2^, respectively) and sea buckthorn seeds oil (500 ng/mL) treatment.

Antioxidant Parameters		Keratinocytes			Fibroblasts		
	Oil	Control	UVA	UVB	Control	UVA	UVB
GSH-Px [mU/mg protein]	-	13.6 ± 0.6	10.8 ± 0.5 ^x^	7.8 ± 0.4 ^x^	11.8 ± 0.6	26.4 ± 1.2 ^x^	29.5 ± 1.4 ^x^
	+	15.6 ± 0.7 ^x^	12.4 ± 0.6 ^ya^	14.8 ± 0.7 ^b^	16.3 ± 0.8 ^x^	26.9 ± 1.3 ^xy^	31.2 ± 1.5 ^xy^
GSSG-R [mU/mg protein]	-	26.3 ± 1.2	23.8 ± 1.2 ^x^	19.2 ± 0.9 ^x^	22.3 ± 1.1	45.6 ± 2.2 ^x^	56.2 ± 2.7 ^x^
	+	22.5 ± 1.1 ^x^	21.8 ± 1.1 ^x^	18.7 ± 0.9 ^xy^	26.9 ± 1.3 ^x^	34.6 ± 1.7 ^xy^	49.3 ± 2.4 ^xy^
SOD [mU/mg protein]	-	28.4 ± 1.3	23.1 ± 1.1 ^x^	21.6 ± 1.1 ^x^	26.3 ± 1.2	21.3 ± 1.0 ^x^	14.6 ± 0.7 ^x^
	+	30.5 ± 1.5	25.6 ± 1.2 ^xy^	24.7 ± 1.2 ^xyb^	20.6 ± 1.0 ^x^	18.5 ± 0.9 ^xya^	12.3 ± 0.6 ^xyb^
TrxR [µU/mg protein]	-	112 ± 5	93 ± 4 ^x^	84 ± 4 ^x^	331 ± 16	204 ± 10 ^x^	141 ± 7 ^x^
	+	104 ± 5	421 ± 11 ^xya^	300 ± 9 ^xyb^	312 ± 15	243 ± 11 ^xya^	182 ± 8 ^xyb^
Trx [µg/mg protein]	-	1.5 ± 0.07	1 ± 0.07 ^x^	0.8 ± 0.07 ^x^	0.9 ± 0.04	0.4 ± 0.01 ^x^	0.4 ± 0.02 ^x^
	+	1.6 ± 0.08	1.6 ± 0.08 ^xa^	1.1 ± 0.07 ^xb^	1.5 ± 0.07 ^x^	1 ± 0.04 ^xya^	1.1 ± 0.05 ^xyb^
GSH [nmol/mg protein]	-	26.2 ± 1.2	11.4 ± 0.5 ^x^	9.6 ± 0.4 ^x^	14.6 ± 0.7	8.2 ± 0.4 ^x^	5.6 ± 0.3 ^x^
	+	37.7 ± 1.8 ^x^	27.7 ± 1.3 ^ya^	22.3 ± 1.1 ^xyb^	15.6 ± 0.8	10.4 ± 0.5 ^xya^	10.1 ± 0.5 ^xyb^
vitamin A [nmol/mg protein]	-	53 ± 2.5	39 ± 1.9 ^x^	35 ± 1.7 ^x^	41 ± 2.1	34 ± 1.6 ^x^	33 ± 1.6 ^x^
	+	63 ± 3.0 ^x^	59 ± 2.8 ^xa^	56 ± 2.7 ^yb^	46 ± 2.2	41 ± 2.1 ^a^	38 ± 1.8 ^xyb^
vitamin E [nmol/mg protein]	-	453 ± 22.1	325 ± 15.9 ^x^	303 ± 14.8 ^x^	336 ± 16.5	287 ±1 4.1 ^x^	269 ± 13.1 ^x^
	+	517 ± 25.3 ^x^	491 ± 24.1 ^ya^	478 ± 23.4 ^yb^	401 ± 19.5 ^x^	381 ± 18.6 ^xa^	351 ± 17.2 ^yb^

Mean values ± SD of five independent experiments are presented. ^**x**^ statistically significant differences vs. control group, *p* < 0.05; ^**y**^ statistically significant differences between UVA/UVB and oil treated groups vs. oil treated control group, *p* < 0.05; ^**a**^ statistically significant differences between UVA and oil treated group vs. UVA treated group, *p* < 0.05; ^**b**^ statistically significant differences between UVB and oil treated group vs. UVB treated group, *p* < 0.05. Abbreviations: UVA: ultraviolet type A; UVB: ultraviolet type B; GSH-Px: glutathione peroxidase; GSSG-R: glutathione reductase; SOD: superoxide dismutase; TrxR: thioredoxin reductase; Trx: thioredoxin; GSH: glutathione.

**Table 4:** The level of phospholipid and free fatty acids in keratinocytes and fibroblasts after exposure to UVA (30 J/cm^2^ and 20 J/cm^2^) and UVB radiation (60 mJ/cm^2^ and 200 mJ/cm^2^, respectively) and sea buckthorn seeds oil (500 ng/mL) treatment.

		Keratinocytes			Fibroblasts		
	Oil	Control	UVA	UVB	Control	UVA	UVB
*Phospholipid fatty acids* [ug/mg protein]							
14:0	-	5.3 ± 0.3	4.5 ± 0.2	4.5 ± 0.2	1.4 ± 0.1	1.2 ± 0.1	1.2 ± 0.1
	+	12.4 ± 0.6 ^x^	11.3 ± 0.6 ^xa^	11.8 ± 0.6 ^xb^	6.3 ± 0.1 ^x^	2.5 ± 0.1 ^xya^	5.1 ± 0.3 ^xyb^
16:0	-	152 ± 7.3	129 ± 6.5 ^x^	128 ± 6.4 ^x^	80 ± 4	68 ± 3.4	68 ± 3.4
	+	662 ± 33.1 ^x^	145 ± 7.3 ^y^	260 ± 13.0 ^xyb^	190 ± 9.5 ^x^	90 ± 4.5 ^ya^	122 ± 6.1 ^xyb^
16:1	-	15.6 ± 0.8	13.2 ± 0.7	13.1 ± 0.7	13.6 ± 0.7	11.6 ± 0.6	11.5 ± 0.6
	+	29.9 ± 1.5 ^x^	27.5 ± 1.4 ^xa^	28.5 ± 1.4 ^xb^	23.2 ± 1.2 ^x^	17.7 ± 0.9 ^xya^	15.9 ± 0.8 ^xyb^
18:0	-	123 ± 6.2	104 ± 5.2 ^x^	103 ± 5.2 ^x^	69 ± 3.5	58 ± 2.9 ^x^	58 ± 2.9 ^x^
	+	412 ± 20.6 ^x^	137 ± 6.9 ^xya^	187 ± 9.4 ^xyb^	126 ± 6.3 ^x^	109 ± 5.5 ^xya^	110 ± 5.5 ^xyb^
18:1nc	-	199 ± 10.1	169 ± 8.5 ^x^	168 ± 8.4 ^x^	112 ± 5.6	95 ± 4.8 ^x^	94 ± 4.7 ^x^
	+	580 ± 29.1 ^x^	129 ± 6.5 ^xya^	386 ± 19.3 ^xyb^	181 ± 9.1 ^x^	167 ± 8.4 ^xa^	114 ± 5.7 ^yb^
18:1nt	-	47 ± 2.4	39 ± 2.1 ^x^	39 ± 2.1 ^x^	18 ± 0.9	16 ± 0.8	15 ± 0.8 ^x^
	+	170 ± 18.5 ^x^	48 ± 2.4 ^ya^	139 ± 7.1 ^xyb^	58 ± 2.9 ^x^	22 ± 1.1 ^xya^	36 ± 1.8 ^xyb^
18:2	-	116.2 ± 5.8	98.8 ± 4.9 ^x^	98.1 ± 4.9 ^x^	45.4 ± 2.3	38.6 ± 1.9 ^x^	38.3 ± 1.9 ^x^
	+	162.7 ± 8.1 ^x^	111.4 ± 5.6 ^ya^	137.2 ± 6.9 ^xyb^	69.8 ± 3.5 ^x^	60.4 ± 3.0 ^xya^	64.5 ± 3.2 ^xb^
18:3n3	-	24.6 ± 1.2	20.9 ± 1.1 ^x^	20.8 ± 1.1 ^x^	6.2 ± 0.3	5.2 ± 0.3	5.5 ± 0.3
	+	29.5 ± 1.5 ^x^	30.2 ± 1.5 ^xa^	31.2 ± 1.6 ^xb^	12.5 ± 0.6 ^x^	7.8 ± 0.4 ^xya^	8.5 ± 0.4 ^xyb^
20:4	-	38.1 ± 1.9	32.4 ± 1.6 ^x^	32.2 ± 1.6 ^x^	35.6 ± 1.8	30.2 ± 1.5	30.1 ± 1.5
	+	197.4 ± 9.9 ^x^	36.4 ± 1.8 ^ya^	104.7 ± 5.2 ^xyb^	81.7 ± 4.1 ^x^	30.3 ± 1.5 ^y^	57.5 ± 2.9 ^xyb^
22:6	-	11.1 ± 0.6	9.4 ± 0.5	9.3 ± 0.5	8.2 ± 0.4	6.9 ± 0.3	6.9 ± 0.3
	+	30.1 ± 1.5 ^x^	18.1 ± 0.9 ^xya^	23.1 ± 1.2 ^xyb^	41.6 ± 2.1 ^x^	16.2 ± 0.8 ^xya^	33.8 ± 1.7 ^xyb^
*Free fatty acids* [µg/mg protein]							
16:0	-	8.8 ± 0.4	7.5 ± 0.4	7.4 ± 0.4	2.9 ± 0.1	2.4 ± 0.1	2.4 ± 0.1
	+	26 ± 1.3 ^x^	14.1 ± 0.7 ^xya^	19.8 ± 1 ^xyb^	9.8 ± 0.5 ^x^	3.9 ± 0.2 ^xya^	7 ± 0.4 ^xyb^
16:1	-	1.7 ± 0.1	1.4 ± 0.1	1.4 ± 0.1	0.7 ± 0.1	0.6 ± 0.1	0.6 ± 0.1
	+	5.5 ± 0.3 ^x^	1.2 ± 0.1 ^y^	4.4 ± 0.2 ^xyb^	2.1 ± 0.1 ^x^	1.3 ± 0.1 ^xya^	1.6 ± 0.1 ^xyb^
18:0	-	7.6 ± 0.4	6.4 ± 0.3	6.4 ± 0.3	3.4 ± 0.2	2.9 ± 0.1	2.9 ± 0.3
	+	16.8 ± 0.8 ^x^	8.8 ± 0.4 ^ya^	8.2 ± 0.4 ^yb^	8.8 ± 0.4 ^x^	3.2 ± 0.2 ^y^	5.5 ± 0.3 ^xyb^
18:1nc	-	13.3 ± 0.7	11.3 ± 0.6	11.2 ± 0.6	3.8 ± 0.2	3.3 ± 0.2	3.5 ± 0.2
	+	54.3 ± 2.7 ^x^	51.1 ± 2.6 ^xa^	45.1 ± 2.3 ^xyb^	6.8 ± 0.3 ^x^	4.1 ± 0.2 ^ya^	5.5 ± 0.3 ^xyb^
18:1nt	-	6.1 ± 0.3	5.2 ± 0.3	5.1 ± 0.3	1.1 ± 0.1	0.9 ± 0.1	0.9 ± 0.1
	+	9.8 ± 0.5 ^x^	8.5 ± 0.4 ^xya^	8.3 ± 0.4 ^xyb^	4.5 ± 0.2 ^x^	1.5 ± 0.1 ^xya^	4.1 ± 0.2 ^xb^
18:2	-	11.7 ± 0.6	9.9 ± 0.5	9.8 ± 0.5	3.2 ± 0.2	2.7 ± 0.1	2.7 ± 0.1
	+	15.1 ± 0.8 ^x^	10.5 ± 0.5 ^y^	12.7 ± 0.6 ^xyb^	5.4 ± 0.3 ^x^	3.3 ± 0.2 ^ya^	3.3 ± 0.2 ^y^
20:4	-	2.2 ± 0.1	1.8 ± 0.1	1.8 ± 0.1	0.7 ± 0.1	0.6 ± 0.1	0.6 ± 0.1
	+	9 ± 0.5 ^x^	5.4 ± 0.3 ^xya^	5.6 ± 0.3 ^xyb^	3.4 ± 0.2 ^x^	0.6 ± 0.1 ^y^	2.7 ± 0.1 ^xyb^
22:6	-	0.9 ± 0.1	0.8 ± 0.1	0.8 ± 0.1	0.3 ± 0.1	0.3 ± 0.1	0.3 ± 0.1
	+	11.3 ± 0.6 ^x^	11.4 ± 0.6 ^xa^	4.8 ± 0.2 ^xyb^	2.3 ± 0.1 ^x^	2.2 ± 0.1 ^xa^	2.2 ± 0.1 ^xb^

Mean values ± SD of five independent experiments are presented. ^**x**^ statistically significant differences vs. control group, *p* < 0.05; **^y^** statistically significant differences between UVA/UVB and oil treated groups vs. oil treated control group, *p* < 0.05; **^a^** statistically significant differences between UVA and oil treated group vs. UVA treated group, *p* < 0.05; **^b^** statistically significant differences between UVB and oil treated group vs. UVB treated group, *p* < 0.05.

## Supplementary Materials

The following are available online at http://www.mdpi.com/2076-3921/7/9/110/s1, Figure S1: The correlation between the level of free fatty acids in sea buckthorn seeds oil and changes in the level of free fatty acids in keratinocytes and fibroblasts after exposure to UVA (30 J/cm^2^ and 20 J/cm^2^) and UVB radiation (60 mJ/cm^2^ and 200 mJ/cm^2^, respectively) and sea buckthorn seeds oil (500 ng/mL) treatment., Figure S2: The electrophorogram images of Western blot analyses of phospho-Nrf2 (pSer40) in keratinocytes and fibroblasts after exposure to UVA (30 J/cm^2^ and 20 J/cm^2^) and UVB radiation (60 mJ/cm^2^ and 200 mJ/cm^2^, respectively) and sea buckthorn seeds oil [500 ng/mL] treatment.
