# Supplementary material for: The Effect of Sea Buckthorn (Hippophae rhamnoides L.) Seed Oil on UV-Induced Changes in Lipid Metabolism of Human Skin Cells
Source: Antioxidants (Basel). 2018 Aug 23;7(9):110. doi: 10.3390/antiox7090110 (PMC6162715; doi:10.3390/antiox7090110)

Supplementary material

Figure S1. The correlation between the level of free fatty acids in sea buckthorn seeds oil and changes in the level of free fatty acids in keratinocytes and fibroblasts after exposure to UVA [30 J/cm^2^ and 20 J/cm^2^] and UVB radiation [60 mJ/cm^2^ and 200 mJ/cm^2^, respectively] and sea buckthorn seeds oil [500 ng/mL] treatment.


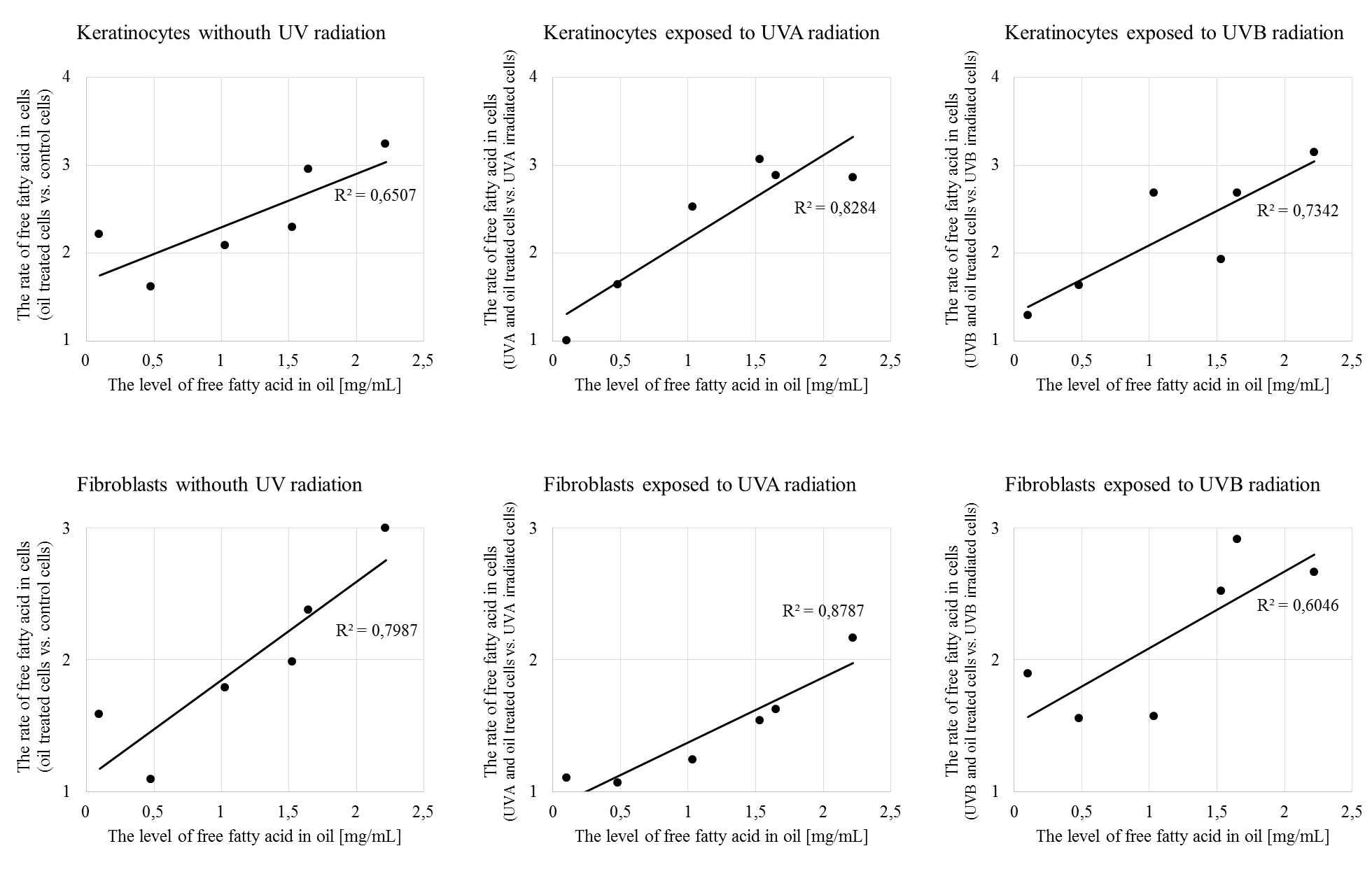


Figure S2. The The electrophorogram images of Western blot analyses of phospho-Nrf2 (pSer40) in keratinocytes and fibroblasts after exposure to UVA [30 J/cm^2^ and 20 J/cm^2^] and UVB radiation [60 mJ/cm^2^ and 200 mJ/cm^2^, respectively] and sea buckthorn seeds oil [500 ng/mL] treatment.


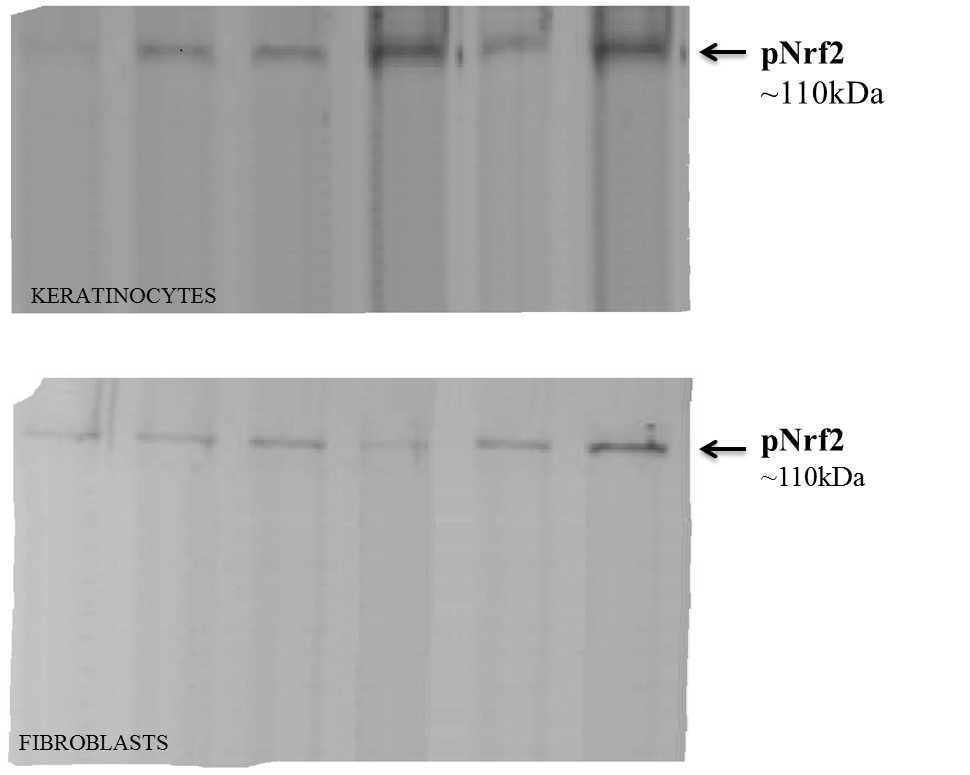

Supplement: Supplementary file 1 [file antioxidants-07-00110-s001.docx]
